# A potential strategy to rebuild the tumor immune microenvironment: PANoptosis

**DOI:** 10.3389/fimmu.2025.1626411

**Published:** 2025-08-04

**Authors:** Siyu Wu, Boyan Tian, Xueying Pang, Bowen Sui

**Affiliations:** ^1^ Heilongjiang University of Chinese Medicine, Harbin, China; ^2^ First Affiliated Hospital, Heilongjiang University of Chinese Medicine, Harbin, China

**Keywords:** PANoptosis, cell death, tumor immune microenvironment, immunotherapy, oncogenic process

## Abstract

The convergence and interplay of pyroptosis, apoptosis, and necroptosis have led to the conceptualization of PANoptosis, an innovative paradigm of inflammatory programmed cell death. Characterized by the hierarchical assembly and activation of the PANoptosome, PANoptosis operates through tightly orchestrated signaling hubs and is intricately linked to organelle functionality. Accumulating evidence underscores its pivotal role in diverse oncogenic processes, positioning PANoptosis as a compelling frontier for antitumor therapeutic exploration. This review delineates the mechanistic underpinnings of PANoptosis, synthesizes its established contributions to tumor progression, and examines its dynamic crosstalk with the tumor immune microenvironment (TIME). Notably, we highlight recent breakthroughs in PANoptosis-driven immunotherapeutic strategies. We further propose that targeting PANoptosis to reprogram TIME represents a transformative approach in oncology, shifting the research paradigm from unimodal cell death regulation to multidimensional intervention. This perspective not only advances fundamental understanding but also holds significant promise for clinical translation, heralding a new era in cancer therapeutics.

## 1 Introduction

Cell death remains a perennial focus in life sciences, broadly categorized into accidental cell death (ACD) and regulated cell death (RCD)—critical feedback mechanisms by which hosts respond to pathogen invasion and external stressors. Among them, RCD involves a series of precise molecular execution and regulation processes, playing an indispensable role in organismal development, damage clearance, and homeostasis maintenance (1). It serves as a critical defense line of the innate immune system. Apoptosis, pyroptosis, and necroptosis represent the most extensively studied RCD pathways, each defined by distinct molecular mechanisms and regulatory networks. Emerging evidence reveals intricate crosstalk among these pathways, forming a dynamic molecular interplay that underpins the novel concept of PANoptosis. PANoptosis exhibits the core features of pyroptosis, apoptosis, and necroptosis but cannot be fully explained by any single pathway alone. When a specific death pathway is selectively blocked, other signaling mechanisms are activated to synergistically enhance alternative pathways, a process regulated by the PANoptosome (2). Currently, the detection of PANoptosis relies on combined experiments using inhibitors/gene knockout and validation of key proteins in the PANoptosome (3).

As a complex and highly coordinated inflammatory RCD pathway, PANoptosis is closely associated with various diseases due to its unique regulatory mechanisms, making it an increasingly prominent topic in modern medical research (4). PANoptosis has emerged as a pivotal player in diverse pathologies, with growing implications in cancer pathogenesis and therapy (5). This review synthesizes the conceptual framework of PANoptosis, highlights its mechanistic basis, and critically evaluates its role across tumor types. We further dissect its interplay with the TIME and explore recent advances in PANoptosis-targeted immunotherapies. By elucidating how PANoptosis reshapes TIME, this work provides a theoretical foundation for novel anticancer strategies, bridging mechanistic insights to translational innovation.

## 2 Overview of PANoptosis

### 2.1 Discovery and definition of PANoptosis

**Figure 1:**
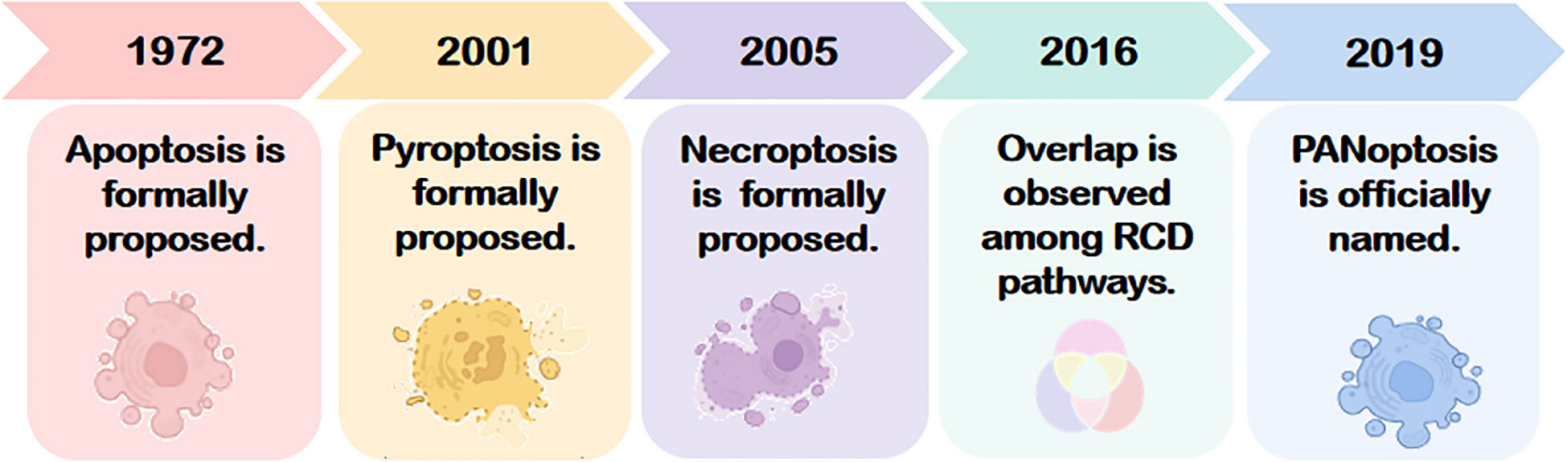
The conceptual evolution of PANoptosis, originating from the discoveries of apoptosis (1972), pyroptosis (2001), and necroptosis (2005). The interconnected mechanism was first demonstrated in 2016, leading to the formal definition of PANoptosis in 2019. RCD, Regulated cell death.

The concept of PANoptosis originated in 2016 from the groundbreaking work of Kanneganti’s team, who demonstrated that influenza A virus (IAV) nucleoprotein and polymerase basic protein 1 activate the cytosolic sensor Z-DNA binding protein 1 (ZBP1). This triggers NOD-like receptors family pyrin domain containing 3(NLRP3)inflammasome and activates the receptor-interacting protein kinase 1(RIPK1)-RIPK3-Caspase-8 signaling axis assembly and synergistically, inducing concurrent apoptosis, necroptosis, and pyroptosis in murine bone marrow-derived macrophages (6). This landmark study unveiled a molecularly interconnected regulatory network among distinct RCD pathways, highlighting mechanistic “overlap” in pathological contexts. In 2019, Kanneganti and colleagues further identified the PANoptosome—a multiprotein complex formed during IAV infection—that serves as a molecular scaffold integrating key effectors of apoptosis, pyroptosis, and necroptosis. This discovery established the spatiotemporal coupling of these three RCD modalities, leading to the formal designation of this convergent death pathway as “PANoptosis” (see **Figure 1**) (7). The term “PAN” reflects the amalgamation of Pyroptosis, Apoptosis, and Necroptosis, as PANoptosis exhibits molecular hallmarks of all three yet cannot be fully recapitulated by any single pathway alone. A deeper exploration of its mechanistic origins is warranted.

### 2.2 Interconnected pathways of apoptosis, pyroptosis, and necroptosis

Apoptosis, pyroptosis, and necroptosis represent the most extensively characterized RCD pathways, each with distinct yet overlapping molecular frameworks. These pathways share a conserved repertoire of interaction domains—including death domains (DDs), caspase recruitment domains (CARDs), and death effector domains (DEDs)—that mediate homotypic or heterotypic interactions to assemble multiprotein complexes, enabling precise regulation of signal transduction and execution.

As a highly ordered and canonical form of RCD, apoptosis was first discovered by Kerr et al. in 1972 and can be initiated via the extrinsic or intrinsic pathways, which require the involvement of death receptors and mitochondria, respectively. Morphologically, it is characterized by preserved membrane integrity, cellular shrinkage, nuclear condensation, and chromatin margination. Challenging traditional views, recent studies have revealed that apoptosis can trigger inflammatory responses through the following mechanisms: (1) Necroptosis pathway: When CASP8 activity is inhibited, RIPK1 escapes cleavage and assembles with RIPK3/FADD into the necrosome, leading to RIPK3-mediated phosphorylation of MLKL and subsequent pro-inflammatory necroptosis; (2) Mitochondrial inflammatory pathway: Under caspase inhibition, mitochondrial outer membrane permeabilization (MOMP) induces degradation of IAP proteins, activating the NF-ĸB pathway and stimulating type I interferon (IFN-1) production; (3) Secondary necrosis pathway: If apoptotic cells are not efficiently cleared, membrane rupture releases damage-associated molecular patterns (DAMPs), triggering an inflammatory storm (8).

In 2001, pyroptosis was first described by Cookson and Brennan as an inflammatory lytic form of RCD mediated by the gasdermin protein family. Orchestrated by the NLRP3 inflammasome, Caspase-1 cleaves Gasdermin D (GSDMD), generating N-terminal fragments that oligomerize to form plasma membrane pores. This disrupts osmotic equilibrium, leading to cell swelling, rupture, and release of pro-inflammatory mediators (e.g., IL-1β, IL-18), a phenomenon termed the “inflammatory cascade” (9).

Necroptosis, first identified in 2005 by Yuan’s team, is a CASP-independent inflammatory RCD (10). Its core mechanism involves sequential activation of receptor-interacting protein kinases (RIPK1, RIPK3) and phosphorylation of mixed-lineage kinase domain-like protein (MLKL), culminating in cell swelling, membrane rupture, and cytoplasmic leakage—hallmarks of necrotic morphology.

**Figure 2:**
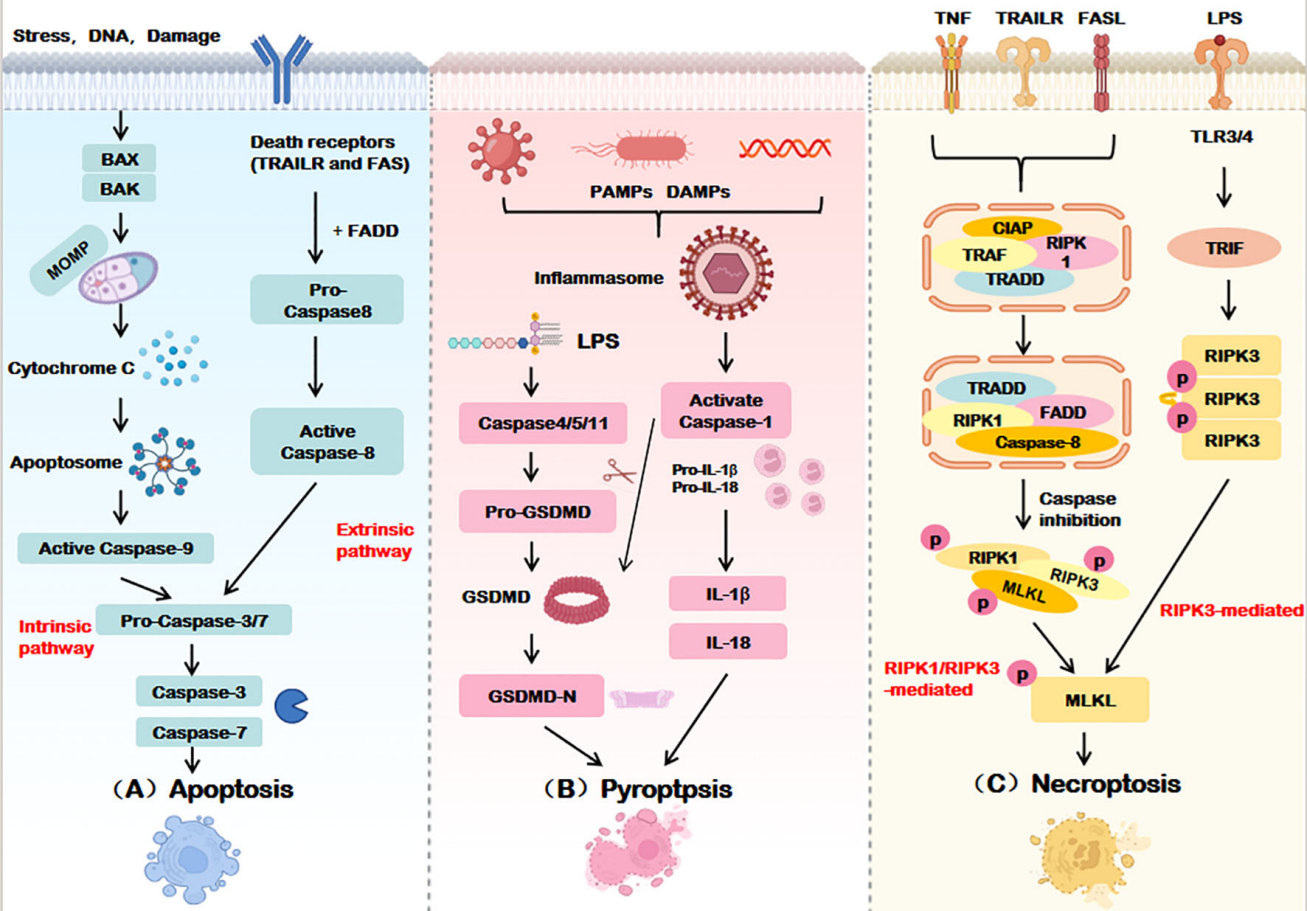
Execution mechanisms of apoptosis, pyroptosis, and necroptosis. **(A)** Apoptosis is mediated by both intrinsic and extrinsic pathways, Intrinsic apoptosis ultimately relies on caspase-9, The extrinsic pathway relies on the activation of caspase-8, Caspase-3/7 is the terminal effector of apoptosis. **(B)** Pyroptosis activates the GSDM family proteins through caspase-1-dependent inflammatosome formation or directly through caspase-4/5/11, forming pores in the cell membrane and inducing cell death. **(C)** Necroptosis phosphorylates MLKL by activating RIPK1 and/or RIPK3 and disrupts the cell membrane to execute death. BAX, BCL2-associated X Protein; BAK, BCL2 Antagonist Killer; MOMP, Mitochondrial outer membrane permeabilization; TRAILR, Tumor necrosis factor-related apoptosis-inducing ligand receptor; FADD, Fas-associated death domain protein; PAMPs, Pathogen-associated molecular patterns; DAMPs, Damage-associated molecular patterns; LPS, Lipopolysaccharide; GSDMD, Gasdermin D; IL-18/1β, Interleukin-18/1β; TNF, Tumor necrosis factor; TLR3/4, Toll-like receptor 3/4; TRIF, TIR-domain-containing adaptor inducing interferon-β; TRADD, Tumor necrosis factor receptor-associated death domain protein; RIPK, Receptor-interacting protein kinase; MLKL, Mixed lineage kinase domain-like.

While apoptosis, pyroptosis, and necroptosis exhibit distinct initiation and execution mechanisms (summarized in **Table 1** and **Figure 2**), their shared molecular architecture underscores a dynamic interplay that drives the emerging paradigm of PANoptosis.

**Table 1:** Distinctive features and interconnections among apoptosis, pyroptosis, and necroptosis.

Feature	Apoptosis	Pyroptosis	Necroptosis
Definition	Programmed cell death, involving a series of ordered biochemical events that eventually lead to the self-destruction of cells.	Inflammatory programmed cell death, usually caused by pathogens or bacterial infections.	Genetically regulated necrotic cell death, activated when apoptosis is inhibited.
Key Triggers	Intrinsic pathway (DNA damage); extrinsic pathway (death receptor activation)	Pathogen infection, inflammasome activation	TNF receptor signaling, viral infection, Caspase-8 inhibition.
Key Effectors	Caspase-3、Caspase-8、Caspase-9、 Bcl-2	Caspase-1、Caspase-4、Caspase-5、 Gasdermin D	RIPK1、RIPK3、MLKL
Morphological Changes	Cell shrinkage, membrane integrity, apoptotic body formation	Membrane pore formation, cell swelling/lysis, inflammatory cytokine release.	Plasma membrane rupture, organelle swelling, necrotic morphology.
DNA Fragmentation	Chromatin condensation, DNA fragmentation, global mRNA decay	Limited DNA damage.	Limited DNA damage.
Inflammatory Response	Non-inflammatory (typically)	Releases IL-1β, IL-18, and DAMPs.	Releases DAMPs (e.g., HMGB1), triggering inflammation.
Physiological Role	Development, tissue homeostasis, removal of damaged cells	Antipathogen defense, immune activation.	Antipathogen defense, inflammatory response
Pathological Role	Excessive apoptosis: tissue atrophy; defective apoptosis: cancer.	Excessive pyroptosis can cause inflammatory diseases	Excessive necroptosis can cause inflammatory disorders (like ischemia-reperfusion injury).

Despite their classification as distinct RCD modalities with unique molecular signatures and morphological hallmarks, emerging evidence highlights significant crosstalk and functional overlap among apoptosis, pyroptosis, and necroptosis. For instance, pyroptosis and necroptosis share a lytic death phenotype, both eliciting inflammatory responses through plasma membrane rupture, albeit via divergent pore-forming mechanisms—Gasdermin proteins in pyroptosis versus MLKL-mediated permeabilization in necroptosis. While pyroptosis and apoptosis both rely on caspase activation, they diverge in effector subtypes: apoptosis is executed by Caspases-3/7, whereas pyroptosis depends on Caspases-1/4/5/11 to cleave GSDMD. Apoptosis and necroptosis intersect at key regulatory nodes, including death receptor signaling (e.g.,TNFR1), stress responses, and shared molecules such as Caspase-8, RIPK1, and RIPK3.

Notably, the caspase and RIPK families play pivotal roles in orchestrating the crosstalk among apoptosis, pyroptosis, and necroptosis. The central function of the caspase family is primarily attributed to caspase-8, which acts as a critical molecular hub mediating the interplay between these three forms of RCD.

When activated, caspase-8 exhibits dual regulatory functions: On one hand, it initiates the extrinsic apoptotic pathway by cleaving caspase-3 and caspase-7, thereby promoting apoptosis. On the other hand, it suppresses necroptosis by proteolytically inactivating RIPK1 and RIPK3. Conversely, in the absence or inhibition of caspase-8, the interaction between RIPK3 and RIPK1 is reinforced, driving MLKL phosphorylation and subsequent necroptosis. Additionally, caspase-8 can cleave GSDMD, thereby triggering pyroptosis (11).

Meanwhile, the RIPK family serves as pivotal molecular switches that dynamically regulate cell fate decisions through conformational changes, functioning as the central processing unit of the RCD regulatory network. Specifically: RIPK1 and RIPK3 interact via their RHIM (receptor-interacting protein homotypic interaction motif) domains to form the necrosome, which mediates necroptotic signaling. RIPK1/3-activated NF-κB signaling upregulates the expression of NLRP3 inflammasome components, thereby promoting caspase-1 activation and pyroptosis. Furthermore, RIPK1, as a critical substrate of caspase-8, undergoes proteolytic cleavage to facilitate the assembly of apoptotic complexes (e.g., the FADD-caspase-8 complex), which activates downstream caspase cascades and drives apoptosis (12).

**Figure 3:**
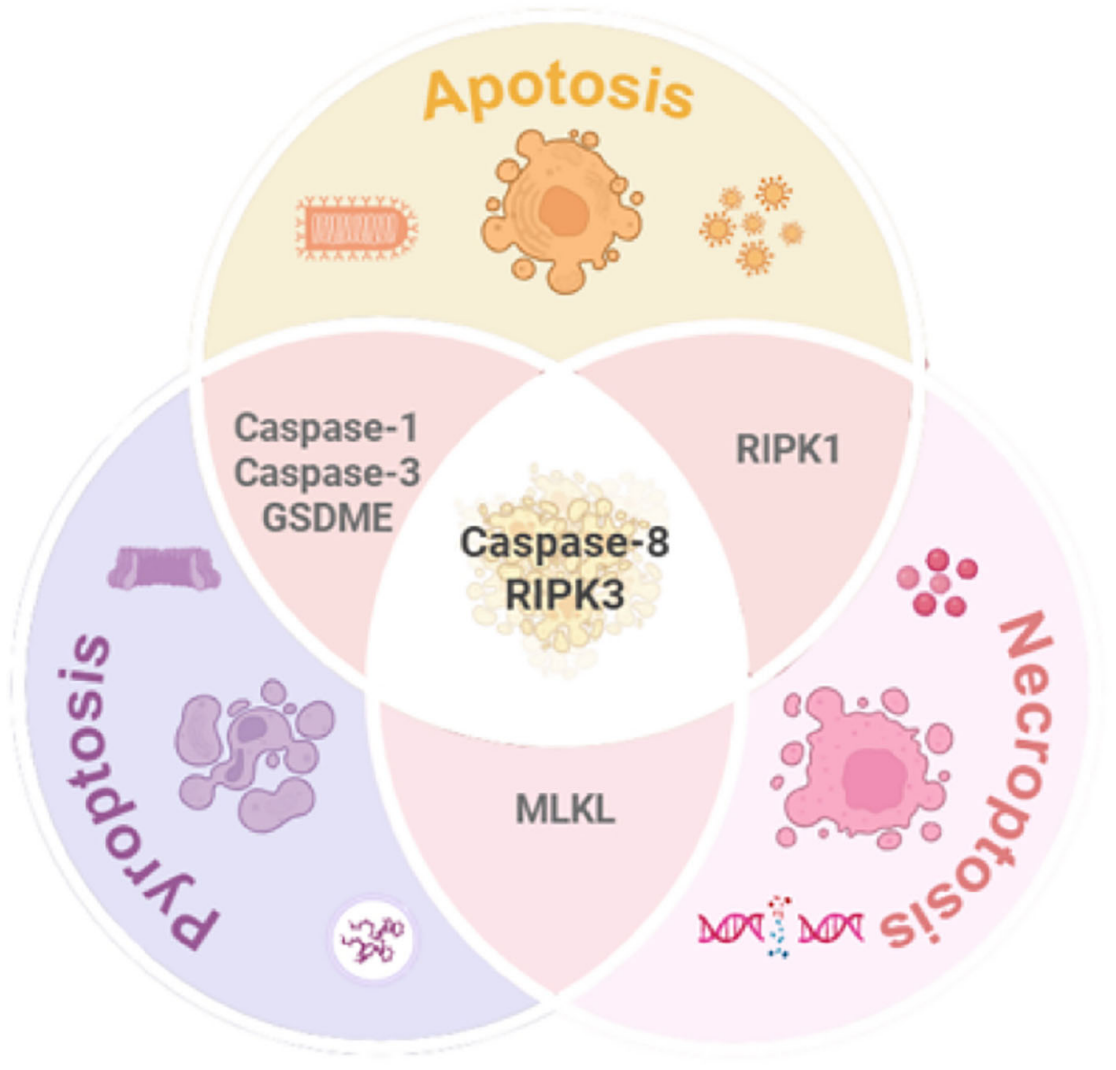
Mechanistic interplay among pyroptosis, apoptosis, and necroptosis. While each pathway exhibits distinct molecular signatures and morphological features, significant crosstalk exists (1): Caspase-1/3 mediates apoptosis-pyroptosis interplay; (2) MLKL serves as a molecular bridge between pyroptosis and necroptosis; (3) RIPK1 complex facilitates dynamic switching between apoptosis and necroptosis. Caspase-8 and RIPK3 emerge as central hubs orchestrating the integration of these three RCD pathways. GSDME, Gasdermin E; MLKL, Mixed lineage kinase domain-like; RIPK1/3, Receptor-interacting protein kinase1/3.

As illustrated in **Figure 3**, these pathways form an intricate regulatory network, where their convergence and redundancy underpin the conceptual framework of PANoptosis. This interconnected architecture enables cells to integrate diverse stress signals, ultimately determining survival or coordinated inflammatory death.

### 2.3 Mechanistic basis of PANoptosis

#### 2.3.1 Assembly of the PANoptosome

PANoptosis is a responsive RCD pathway initiated by viral or bacterial infection, or upstream molecular signaling cascades, which drives the assembly of the multiprotein PANoptosome complex. This scaffold activates downstream RCD executors, enabling the integration and spatiotemporal regulation of pyroptosis (via inflammasomes), apoptosis (via apoptosomes), and necroptosis (via necrosomes). The PANoptosome facilitates signal transduction and crosstalk among these pathways through dynamically reconfigured components, serving as the central hub for PANoptotic regulation. Genetic and proteomic studies confirm that the PANoptosome comprises three core modules: sensor proteins, adaptors, and effector proteins.

##### 2.3.1.1 Sensor module

PANoptosis is triggered by pattern recognition receptors (PRRs) sensing pathogen-associated molecular patterns (PAMPs) or damage-associated molecular patterns (DAMPs) (4). Key sensors include ZBP1, RIPK, and inflammasome sensors. ZBP1, a cytosolic innate immune sensor of viral infection, mediates cell death and inflammation via its Zα2 domain, which is essential for inflammasome activation and PANoptosis (13). It also acts as an apical sensor in fungal infection-induced PANoptosis (14). RIPK1, critical for *Yersinia*-induced PANoptosis, recruits ZBP1, Caspase-8, and other factors to assemble the PANoptosome through homotypic or heterotypic interactions (15). Inflammasome sensors such as Absent in melanoma 2(AIM2), NLRP3, NLRC4, and Pyrin are integral PANoptosome components that drive inflammatory death (16). For example, AIM2 detects cytoplasmic double-stranded DNA released during pathogen invasion or cellular stress, promoting inflammasome assembly and PANoptosome activation (17). NLRP12 functions as a cytoplasmic sensor in heme- and PAMP-driven PANoptosis (18), while NLRC5 interacts with NLRP12 and other PANoptosome components to form death complexes (19).

##### 2.3.1.2 Adaptor module

Adaptor proteins, including apoptosis-associated speck-like protein (ASC) and Fas-associated death domain protein (FADD), bridge sensor and effector modules. These scaffold molecules mediate protein-protein interactions to propagate PANoptotic signals.

##### 2.3.1.3 Effector module

Effectors such as caspases, RIPK1/RIPK3 kinases, and MLKL execute PANoptosis through proteolytic cleavage, phosphorylation cascades, or membrane permeabilization (20). Caspase-1 and Caspase-8 exhibit multifunctional roles in PANoptosis (2), while Caspase-6 enhances ZBP1-RIPK3 interactions to promote PANoptosome assembly, though its precise activation mechanism remains elusive (21).

**Figure 4:**
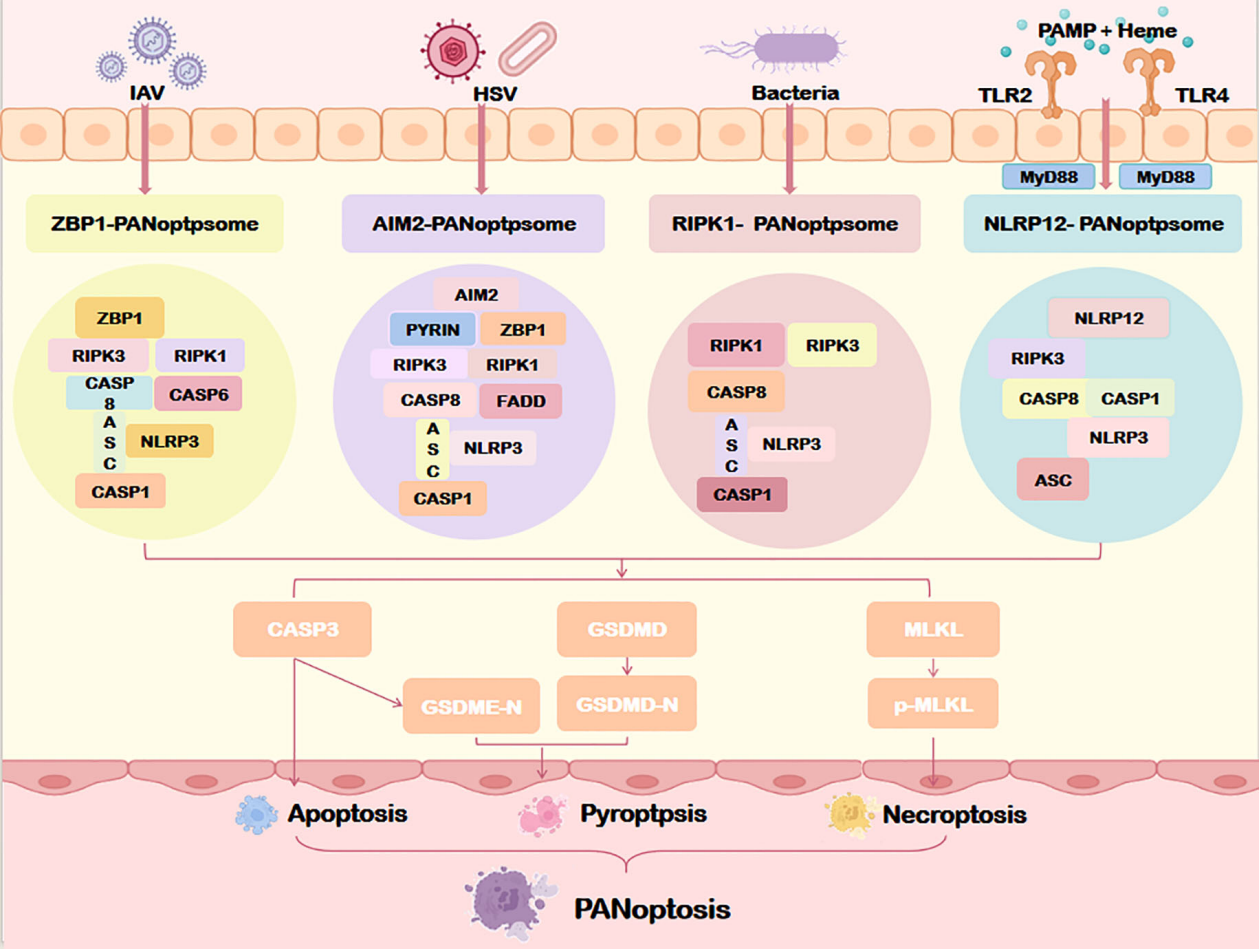
Assembly of the PANoptosome. Specific sensors (including ZBP1, AIM2, RIPK1, and NLRP12) form distinct PANoptosome complexes that differentially regulate downstream PANoptosis pathways through specialized molecular mechanisms. IAV, Influenza a virus; HSV, Herpes simplex virus; MyD88, myeloid differentiation factor 88; TLR2/4, Toll-like receptor 2/4; ZBP1, Z-DNA binding protein 1; AIM2, Absent in melanoma 2; NLRP1/12, Nucleotide-binding oligomerization domain, leucine rich repeat and pyrin domain containing protein 1/12; ASC, Apoptosis-associated speck-like protein containing a card; CASP1/3/8, Caspase1/3/8. Other abbreviations are the same as **Figure 2**.

Four PANoptosome subtypes have been identified to date: ZBP1-dependent, AIM2-mediated, RIPK1-regulated, and NLRP12-associated complexes (**Figure 4**). These subtypes reflect context-specific adaptations to diverse pathogenic or stress stimuli.

#### 2.3.2 Organellar regulation of PANoptosis

Targeting mitochondrial dysfunction represents a potential strategy to mitigate PANoptosis. As the cellular “powerhouse,” mitochondrial impairment is a key pathological driver of PANoptosis. Studies reveal that defects in mitochondrial complex I provoke mtDNA release, which activates ZBP1-dependent PANoptosis (22). Additionally, mtDNA activates the cGAS-STING axis, exacerbating PANoptosis-associated cell death (23, 24). Mitochondrial ROS (mtROS) accumulation further promotes PANoptosome assembly, with mtROS scavengers shown to suppress PANoptosis (25).

Lysosomal dysregulation also contributes to PANoptosis. Aberrant lysosome-associated membrane protein 2A (LAMP2A) pathways in neurons act as early inducers of PANoptotic signaling and neuroinflammation, driving post-ischemic stroke neurological deficits alongside upregulation of FADD, RIPK3, and MLKL (26).

The ER central to protein and lipid synthesis, plays a critical role in PANoptosis. Under deacetylation conditions, malate dehydrogenase 1 (MDH1) and isocitrate dehydrogenase 1 (IDH1) activate ER stress signaling, amplifying PANoptosis in acute liver failure (27). These findings underscore the multisystemic integration of organellar stress in PANoptotic regulation.

#### 2.3.3 Molecular switches governing PANoptosis

The progression of PANoptosis is tightly regulated by upstream signaling pathways, with the interferon (IFN) axis emerging as a pivotal orchestrator. Interferon Regulatory Factor 1 (IRF1), a master transcriptional regulator of IFN signaling, acts as a molecular switch by driving the expression of innate immune genes critical for PANoptosis initiation (28). For instance, during influenza A virus (IAV) infection, IRF1 upregulates ZBP1 to facilitate ZBP1-PANoptosome assembly, positioning ZBP1 as a sensor of viral ribonucleoproteins that triggers macrophage PANoptosis (29). IRF1 also modulates AIM2 inflammasome activity (30) and activates the NLRP12-PANoptosome in hemolytic diseases (18). In colorectal cancer, IRF1 exerts tumor-suppressive effects by enhancing PANoptosis (31). During SARS-CoV-2 infection, TNF-α and IFN-γ synergistically induce PANoptosis via the JAK-IRF1 axis (32), while IRF1 itself can paradoxically suppress TNFα/IFNγ-driven PANoptosis in renal endothelial cells, underscoring its context-dependent regulatory duality (33). Collectively, these findings establish IRF1 as a central molecular switch in PANoptotic networks.

Transforming growth factor beta-activated kinase 1 (TAK1), a core MAPK pathway regulator, serves as another critical switch (34). TAK1 inhibition enhances RIPK1 autophosphorylation, promoting assembly of the ASC/Caspase-8/RIPK3 complex to drive PANoptosis (35). Kongensin A, a natural compound, suppresses PANoptosis by upregulating TAK1 to maintain mitochondrial redox balance, validating TAK1’s role as a gatekeeper of PANoptosome assembly (36). CRISPR screens further identified PTBP1, RAVER1, and as modulators of TAK1 inhibitor-induced PANoptosis, with their depletion blunting RIPK1-mediated death (37). In addition, the Protein phosphatase 6 (PP6) holoenzyme is also a regulatory factor of TAK1I-induced PAN-apoptosis (38).

In this part, we systematically elucidate the mechanisms, distinctions, and interconnected relationships among apoptosis, pyroptosis, and necroptosis, providing an in-depth exploration of the theoretical origin and core concept of PANoptosis. PANoptosis is executed through the cascade assembly and dynamic activation of a multiprotein complex—the PANoptosome. Its molecular regulatory network not only involves the precise modulation of key signaling hub molecules but also deeply integrates the functional states of organelles such as mitochondria, lysosomes, and the endoplasmic reticulum. Clarifying the structural basis and mechanistic framework of PANoptosis establishes a theoretical foundation for subsequent investigations into its roles in tumorigenesis, progression, and immunotherapy.

## 3 PANoptosis in tumorigenesis

**Figure 5:**
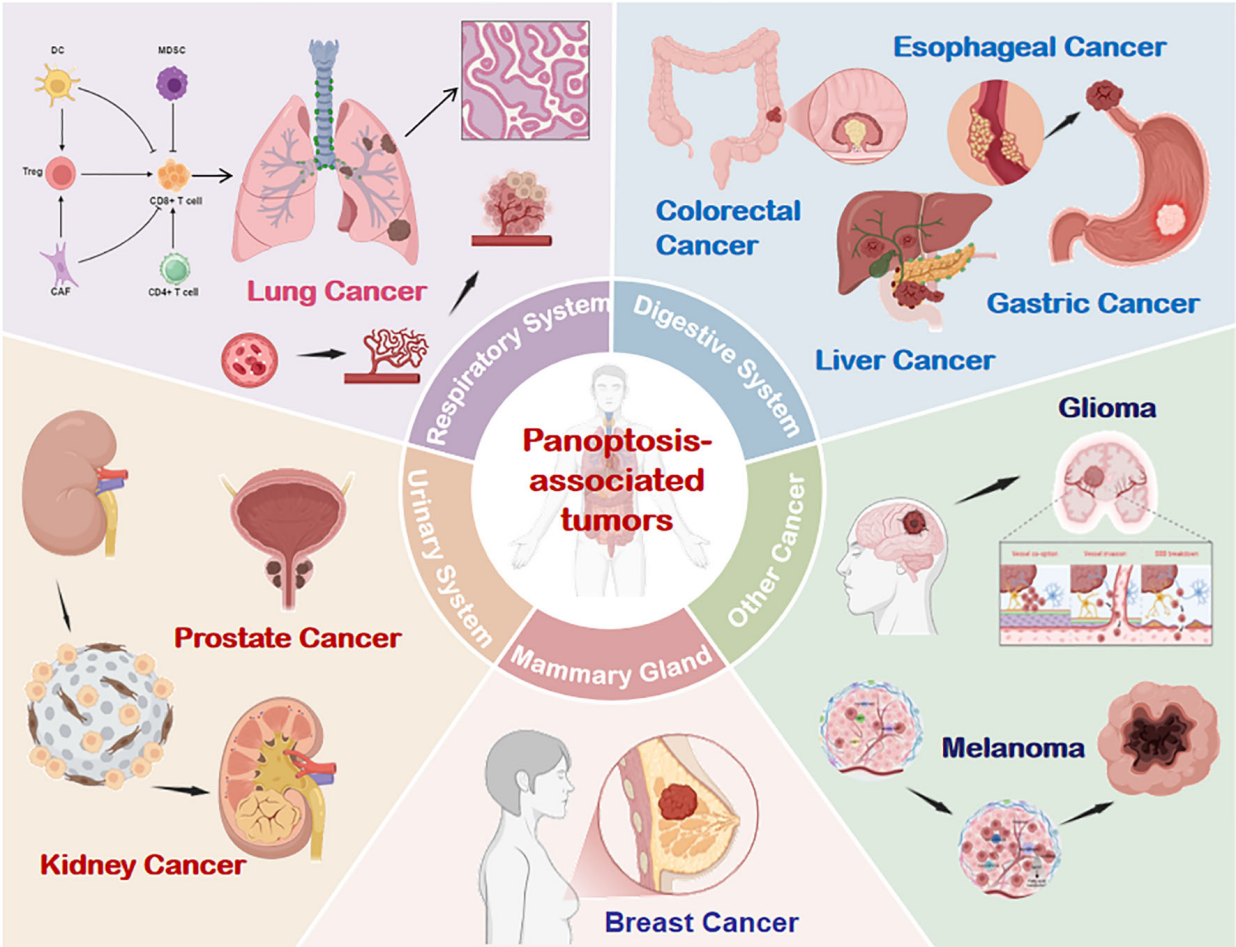
PANoptosis involvement across multiple tumor types. Emerging evidence confirms the presence and functional significance of PANoptosis in respiratory, digestive, urinary, breast, and other cancer systems. DC, dendritic cells; CAF, Cancer-Associated Fibroblasts; NK, natural killer; Treg, Regulatory T cell; MDSC, Myeloid-Derived Suppressor Cells.

The role of PANoptosis in cancer is complex and multifactorial (39). On one hand, it constrains tumorigenesis by eliminating damaged or malignant cells to maintain intratumoral homeostasis (40, 41). Conversely, hyperactivation of PANoptosis may drive immunocyte death, fuel inflammatory cascades, and foster immunosuppressive microenvironments that facilitate tumor immune evasion and progression (42). While mechanistic details remain incompletely mapped, empirical evidence underscores its involvement across diverse malignancies. (**Figure 5**).

Lung cancer remains a leading cause of global cancer morbidity and mortality, as highlighted by 2020 Global Burden of Disease data (43). FADD, a core PANoptosis adaptor, exhibits nucleocytoplasmic shuttling linked to lung carcinogenesis. Wei et al. demonstrated elevated FADD expression in lung adenocarcinoma (LUAD) tissues compared to adjacent normal tissue, correlating with poor patient survival (44). *In vitro* suppression of FADD reduced the proliferation of lung cancer cells and altered expression of apoptosis (BAX/BCL-2) and pyroptosis (Caspase-1/NLRP3) markers, suggesting that regulating pan-apoptotic markers may potentially become therapeutic targets for lung adenocarcinoma.

Gastric cancer (GC) is linked to PANoptosis. Dysregulated long non-coding RNAs (lncRNAs) are implicated in GC progression. Hong et al. constructed a prognostic model based on PANoptosis-related lncRNAs (PANlncRNAs), identifying candidates linked to GC prognosis, chemotherapy response, and immune infiltration, offering potential biomarkers and therapeutic targets (45). Lin et al. revealed that Y-box binding protein 1 (YBX1) suppresses PANoptosis to enhance oxaliplatin resistance in GC cells (46). Mechanistically, ubiquitin-specific protease 10 (USP10) collaborates with protein phosphatase 1B (PPM1B) to degrade YBX1, inducing PANoptosis and overcoming chemoresistance—a finding with translational implications.

Esophageal cancer (EC) is increasingly linked to PANoptosis. Fu et al. identified PANoptosis-related genes (PRGs) through bioinformatics analysis, constructing an eight-gene prognostic model that stratifies EC patients by survival outcomes, immune microenvironment features, and drug sensitivity (47).

Hepatocellular carcinoma (HCC) is the most common type of primary liver cancer. Qi et al. developed a PRG-based prognostic model (PRL) for HCC, with lncRNA AC026412.3 showing superior predictive power (48). Knockdown of AC026412.3 elevated Caspase-3, pro-apoptotic Bax, NLRP3 inflammasome, and phosphorylated MLKL, mechanistically linking PANoptosis activation to tumor suppression.

Colorectal cancer (CRC), the third most prevalent and second deadliest malignancy globally, poses a major public health challenge (43). A multi-omics analysis of 458 CRC patients identified PLCB2, CAV1, and DAPK1 as PANoptosis-interacting genes and defined two genetic subtypes, underscoring PANoptosis-immune crosstalk in CRC progression (49).

Breast cancer (BC), the leading cause of cancer-related morbidity in women, exhibits declining mortality but rising incidence due to disparities in screening and prevention (50). Qian et al. delineated PANoptosis triggers and signaling cascades in BC, informing novel targeted therapies (51). Wang et al. integrated single-cell sequencing and machine learning from 6,900 BC patients to identify six PANoptosis hub genes (CD24, BMF, DAPK2, GNAI3, NR4A2, SRC), constructing a prognostic model to guide combination therapies (52).

Adrenocortical carcinoma (ACC), a rare and aggressive malignancy, is linked to PANoptosis. Ren et al. identified cyclin-dependent kinase 1 (CDK1) overexpression as a predictor of poor ACC outcomes (53). *In vitro*, the CDK1 inhibitor cucurbitacin E (CurE) upregulated apoptosis (Caspase-3/7), pyroptosis (Caspase-1/GSDMD), and necroptosis (RIPK1/RIPK3/MLKL) markers in ACC cells. ZBP1 knockdown abolished these effects, confirming CurE-induced ZBP1-dependent PANoptosis.

In prostate adenocarcinoma (PRAD), Yi et al. mapped PANoptosis pathway perturbations—including mutations, transcriptional dysregulation, and methylation—and correlated them with clinical features to predict prognosis and immunotherapy response (54).

Chrysoeriol, a natural flavonoid, has the potential to inhibit melanoma. Liu et al. found that the morphological changes of melanoma cells treated with chrysoeriol showed features related to PANoptosis and ferroptosis. Chrysoeriol can not only induce apoptosis, alter mitochondrial membrane potential, increase ROS production, and promote necroptosis, but also upregulate molecules related to pyroptosis and ferroptosis (55).

In glioma, Chen et al. classified tumors into PANoptosis-related gene clusters and developed a machine learning-based artificial neural network model to predict prognosis, highlighting PANoptosis’ role in neuro-oncology (56).

## 4 PANoptosis and TIME

Tumor microenvironment (TME), a dynamic and highly complex ecosystem, provides a permissive “soil” for tumor cell growth and progression (57). Based on cellular composition, the TME can be further classified into distinct subtypes, among which TIME centered on immune cells is of particular importance. To date, targeting the TIME to enhance host antitumor immune responses has emerged as a key research focus in cancer therapeutics (58).

**Figure 6:**
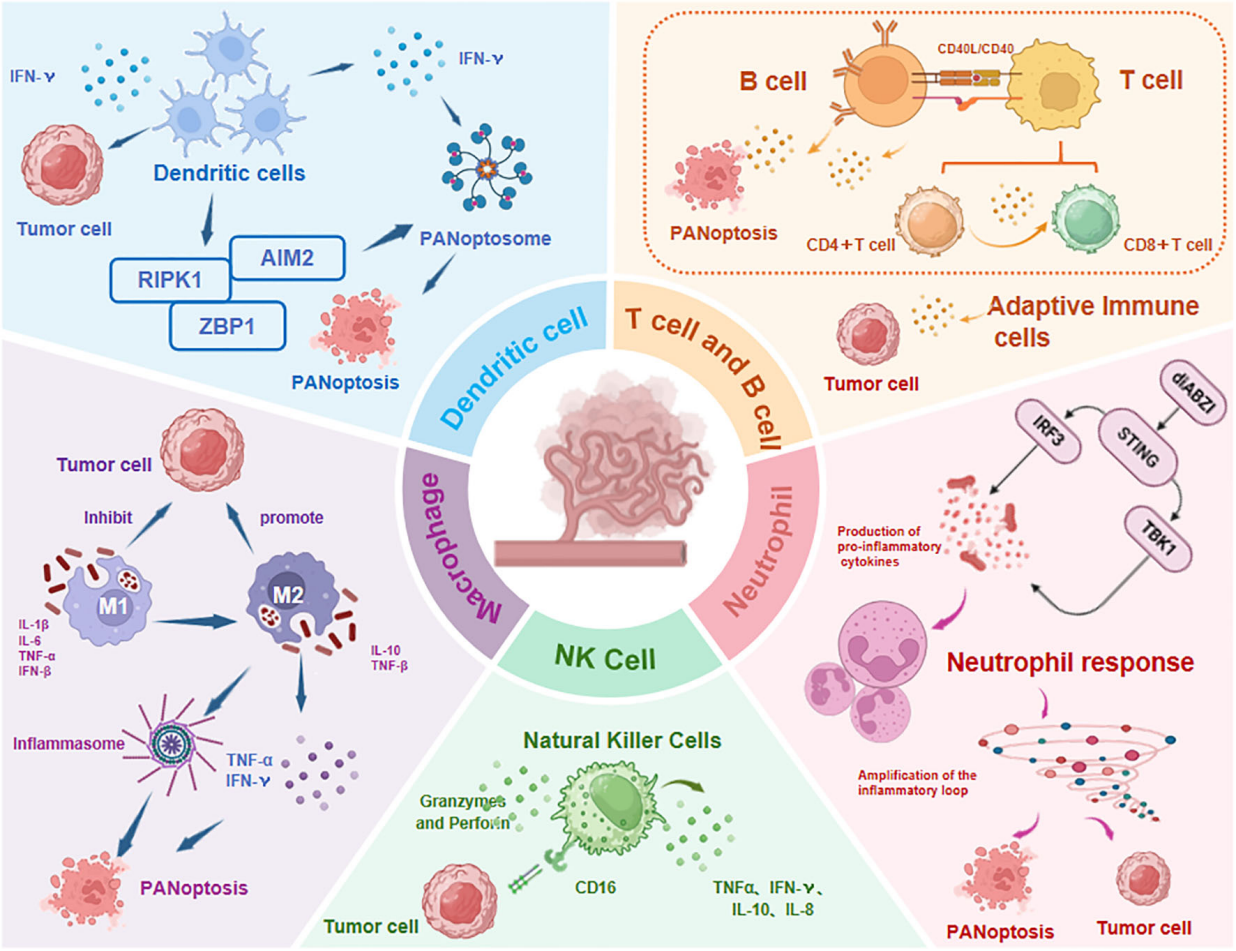
Immune cell dynamics in TIME regulated by PANoptosis. The infiltration levels and functional states of both innate immune cells (NK cells, macrophages, TANs, DCs) and adaptive immune cells (T/B lymphocytes) show significant correlation with PANoptosis activity. TNF-α, Tumor Necrosis Factor-α; IFN-γ, Interferon-γ; diABZI, dimeric Amidobenzimidazole; STING, Stimulator of interferon genes; IRF3, Regulatory Factor 3; TBK1, TANK-Binding Kinase 1; Abbreviations not mentioned are the same as those in other figures.

As a markedly immunogenic form of RCD, PANoptosis engages with the TIME through intricate crosstalk by driving innate immunity and inflammatory responses (12). Accumulating evidence indicates that the infiltration levels and functional states of innate immune cells (e.g., NK cells, macrophages, tumor-associated neutrophils TANs, and dendritic cells DCs) and adaptive immune cells (e.g., T and B cells) within the TIME are closely associated with PANoptotic activity in tumors. The current evidence is reviewed below (**Figure 6**).

### 4.1 PANoptosis and innate immune cells

Innate immune cells, the body’s first-line defense, provide rapid and broad-spectrum responses to pathogens and malignancies. Key players include natural killer (NK) cells, macrophages, neutrophils, and dendritic cells (DCs).

#### 4.1.1 PANoptosis and natural killer cells

NK cells, cytotoxic effectors of innate immunity, eliminate tumor cells via granzyme/perforin-mediated cytotoxicity, Fas ligand (FasL)-induced apoptosis, and IFNγ production, while orchestrating adaptive immune responses (59). Emerging evidence implicates NK cells in PANoptosis-driven antitumor immunity. A pan-cancer analysis revealed significant correlations between PANoptosis-related gene signatures and NK cell infiltration within TIME (60). In HCC, intratumoral and circulating NK cells correlate with improved survival (61).

Mechanistically, RIPK3 and RIPK1 bridge PANoptosis to NK cell-mediated immunity. RIPK3 expression positively correlates with NK cell infiltration, and its activation enhances antitumor immunity in TME (62). Gong et al. found that RIPK1-mediated RCD can increase the infiltration of NK cells in TIME and enhance the survival benefit of immune checkpoint blockade (63). Bioinformatics studies identified PANoptosis-associated genes (NFKBIA, RNF34, SERINC3) linked to CD56dimdim NK cell infiltration (64), while machine learning-derived PRGs (BIRC3, MAGED1, PSME2) further associate with NK cell enrichment (65). Though evidence remains nascent, these findings collectively establish NK cells as pivotal mediators of PANoptosis-immune crosstalk in cancer.

#### 4.1.2 PANoptosis and macrophages

Macrophages, derived from bone marrow monocytes, constitute over 50% of tumor-infiltrating immune cells and exhibit functional plasticity between pro-inflammatory M1-like (anti-tumor) and immunosuppressive M2-like (pro-tumor) phenotypes. M1 macrophages secrete IL-1β, IL-6, TNF-α, and IFN-β to drive antitumor immunity, while M2 macrophages promote angiogenesis and immune evasion (66).

Li et al. identified PANoptosis-related differentially expressed genes in thyroid cancer, suggesting its role in M1-to-M2 macrophage polarization during tumorigenesis (67). TNF-α, a key M1-derived cytokine, activates PANoptosis to eliminate cancer cells, as demonstrated by Malireddi et al. (68). In cervical cancer, Qiang et al. linked immune subtype-associated recurrence risk scores to PANoptosis, emphasizing macrophage-driven TIME regulation (69). Cinobufagin, a PANoptosis-inducing agent, reprograms macrophages toward M1-like phenotypes, enhances CD4^+^/CD8^+^ T cell infiltration, and improves glioma outcomes (70). These findings underscore macrophages as both responders to and regulators of PANoptosis-modulated TIME.

#### 4.1.3 PANoptosis and dendritic cells

DCs, specialised antigen-presenting cells of the innate immune system, orchestrate adaptive immunity by priming T-cell responses. While direct DC-PANoptosis interactions remain uncharacterized, DCs are implicated in PANoptosis regulation. In pancreatic cancer, Zhang et al. demonstrated that DC-enriched tumors exhibit heightened sensitivity to erlotinib, selumetinib, and trametinib (71). Zhou et al. proposed that PANoptosis generates immunogenic tumors via DAMP release, promoting DC maturation and macrophage polarization to reprogram immunosuppressive TIME and enhance antitumor immunity (72). IFN-γ, a key DC-secreted cytokine, is critical for PANoptosis induction in murine models (73). IFN-γ deficiency impairs activation of PANoptosis markers (e.g., CASP3, GSDMD, MLKL) and reduces IL-1β production (74). Notably, TNF-α and IFN-γ synergize to activate GSDMD, GSDME, Caspases (-3/-7/-8), and MLKL, triggering PANoptosis across 13 human cancer cell lines (including colon, lung, melanoma, and leukemia), highlighting cytokine-driven PANoptosis as a therapeutic target (68). DCs facilitate PANoptosome assembly by upregulating cytosolic innate sensors (e.g., ZBP1, AIM2, RIPK1) and regulatory factors, thereby driving PANoptosis initiation (2). This positions DCs as pivotal modulators of PANoptosis-immune crosstalk in cancer.

#### 4.1.4 PANoptosis and neutrophils

Neutrophils, constituting 50–70% of human and 10–25% of murine circulating leukocytes, serve as frontline defenders against pathogens (75). In the TME, tumor-associated neutrophils (TANs) exhibit context-dependent antitumor or protumor functions (76). TANs exhibit remarkable plasticity and functional diversity within TIME. Based on their roles, TANs are categorized into distinct subtypes: N1-like TANs exert antitumor effects by facilitating antigen presentation, recruiting and activating T cells. N2-like TANs promote tumor progression via extracellular matrix (ECM) remodeling, angiogenesis, tumor cell migration, and immune evasion. N0-like TANs represent a transitional state between N1 and N2 phenotypes, retaining functional ambivalence (77). Stimulator of interferon genes (STING), a key PANoptosis inducer, drives neutrophil activation, neutrophil extracellular trap (NET) formation, and dsDNA release upon stimulation with the STING agonist diABZI, culminating in STING-dependent PANoptosis (23). In non-small cell lung cancer (NSCLC), Hu et al. identified a protumor TAN subset overexpressing high-mobility group box 1 (HMGB1), a chromatin protein that impairs neoantigen presentation and suppresses antitumor immunity. HMGB1-positive TANs exhibit enriched PANoptosis-related gene expression and promote immune evasion via the GATA2/HMGB1/TIM-3 axis (78). These findings position TANs as dynamic regulators of PANoptosis-immune interplay in cancer.

### 4.2 PANoptosis and adaptive immune cells

Adaptive immunity, characterized by antigen-specific responses and immunological memory, is mediated by T and B lymphocytes. T cells—including cytotoxic CD8^+^ and helper CD4^+^ subsets—orchestrate cellular immunity and tumor cell killing, while B cells eliminate malignancies via antibody production, cytokine secretion, and antigen presentation.

T/B cell-PANoptosis interplay is increasingly evident across cancers. In pancreatic cancer, Zhang et al. linked PANoptosis to heightened CD8^+^ T cell and naïve B cell infiltration, correlating with sensitivity to irinotecan, oxaliplatin, and sorafenib (71). Gao et al. demonstrated that T cell-derived IFN-γ triggers PANoptosis alongside antitumor immunity (5), while Cai et al. developed a PANoptosome assembly potential index (PANo-RPI), showing high PANo-RPI correlates with CD8^+^ T cell infiltration (79). Li et al. implicated PANoptosis in thyroid cancer immune dysregulation via modulation of activated T/B cells and TNF signaling (67). Hou et al. engineered hydrazide hyaluronic acid-coated Zn-CuO_2_ nanoparticles to induce PANoptosis, amplifying CD8^+^ T cell-mediated immunity (80). In diffuse large B-cell lymphoma (DLBCL), Xu et al. developed the PANoptosis-related gene prognostic index (PANGPI) based on diffuse large B-cell lymphoma (DLBCL). The study found that the infiltration level of CD8^+^ T cells was positively correlated with the PANGPI risk score, while CD4^+^ T cells and NK cells were negatively correlated with the PANGPI risk score. In addition, the study revealed that chemotherapy drugs (BMS-536924, gefitinib, navitoclax) were used to treat high-risk DLBCL patients (81). These studies collectively validate PANoptosis as a nexus of adaptive immune regulation, offering novel biomarkers and therapeutic strategies to rebuild TIME.

## 5 PANoptosis-based cancer immunotherapy

Targeting TIME to augment host antitumor immunity has emerged as a pivotal strategy in cancer therapeutics. As outlined above, PANoptosis—an innate immune-mediated inflammatory programmed cell death pathway—plays a multifaceted role in tumor initiation, progression, and metastasis. Its intricate interplay with TIME, particularly through modulating immune cell infiltration and functional states, critically shapes the efficacy of antitumor immune responses. In contrast to individual forms of RCD, the core advantage of PANoptosis lies in its ability to integrate and activate multiple cell death pathways, thereby generating potent and sustained immunogenic effects. This unique characteristic renders PANoptosis irreplaceable in the field of cancer immunotherapy.

Therefore, this review proposes that remodeling the TIME through PANoptosis represents an emerging strategy for cancer immunotherapy to suppress tumor progression. However, its clinical translation remains exploratory. Nevertheless, current studies have demonstrated that modulating PANoptosis can effectively overcome key limitations of immunotherapy. This section systematically evaluates the therapeutic potential of PANoptosis in antitumor immunity and provides a theoretical framework for TIME reprogramming.

As previously discussed, researchers are actively leveraging the molecular signatures of PANoptosis to develop predictive tumor models and identify potential therapeutic targets for cancer treatment. Notably, Cai et al. demonstrated that a high PANo-RPI shows significant positive correlation with improved immunotherapy response rates and clinical outcomes. Their study suggests that pharmacologically targeting PANoptosis in cancer cells may serve as an effective strategy to convert immunologically “cold” TMEs into “hot” TMEs, thereby potentiating antitumor immunity (79). In HCC, bioinformatic studies linked PANoptosis to patient survival and immune responsiveness, identifying BAK1 and CSE1L as differentially expressed prognostic genes (82). Similarly, the HCC PANoptosis index (HPAN) stratifies patients into high- and low-response subgroups, with high HPAN associated with elevated PANoptosis gene expression and superior immunotherapy outcomes (83). These models underscore the clinical relevance of PANoptosis in predicting immune reactivity and guiding therapeutic stratification.

Key regulators and components of PANoptosis represent promising targets for cancer immunotherapy. The Caspase family, central to PANoptosis execution, has emerged as a critical focus. Lou et al. demonstrated that Caspase-2 modulates epithelial-mesenchymal transition (EMT) signaling in HCC, reshaping TIME and influencing immunotherapy response rates (84). Caspase-8, a “molecular switch” in PANoptosis, governs tumorigenesis and TIME dynamics, with its targeting offering potential to reprogram immunosuppressive microenvironments (85). Chen et al. further identified Caspase-8 as a therapeutic node in HCC, linking PANoptosis, inflammation, and TME remodeling (86).

NLRC5, a polymorphic innate sensor, drives PANoptosis in response to pathogen-associated molecular patterns (PAMPs) or heme/cytokine combinations. Sundaram et al. revealed that NLRC5 collaborates with NLRP12 to form the NLRC5-PANoptosome complex, regulating inflammatory cell death and tumor progression (19). This positions NLRC5 as a dual regulator of immunity and inflammation, with therapeutic potential in cancer.

PANoptosis addresses drug resistance and synergizes with conventional immunotherapies. The application of PANoptosis in overcoming therapeutic resistance is gaining momentum (87). For instance, SNHG7 correlates with chemoresistance in colon adenocarcinoma (88), while NFS1 deficiency enhances oxaliplatin sensitivity in colorectal cancer (89). Sulconazole induces PANoptosis in esophageal cancer by triggering oxidative stress and glycolytic inhibition, thereby improving radiosensitivity (90). Notably, PANoptosis—by integrating three RCD modalities—elicits robust antitumor immunity. Combining PANoptosis inducers with immunotherapies enhances treatment efficacy by upregulating immune checkpoint expression and subverting immunosuppressive TIME (87). PANoptosis elevates PD-L1 levels, potentiating T-cell activity when paired with immune checkpoint inhibitors (ICIs) (87, 91). Additionally, PANoptosis disrupts immunosuppressive niches by depleting regulatory T cells (Tregs) and suppressing TGF-β production, thereby curtailing tumor progression (87).

Currently, nanoengineered approaches targeting PANoptosis remain in the exploratory stage with limited coverage, yet emerging evidence suggests this strategy may evolve into a novel therapeutic paradigm for antitumor immunotherapy. Ultrasound-responsive nanomedicine, for example, employs engineered extracellular vesicles to trigger immunogenic PANoptosis. This approach activates cyclic innate immune responses via DAMP release, primes tumor-specific T cells, and amplifies antitumor immunity through cGAS-STING pathway activation (92, 93). Ma et al. (94)developed an integrated approach combining photodynamic therapy and chemodynamic therapy within nanoliposomes. They utilized the photosensitizer Chlorin e6 to generate reactive oxygen species and the natural targeting agent Jolkinolide B to activate PANoptosis molecular switches. This strategy effectively induced ROS-caspase8/PANoptosis pathway-mediated GC cell death. This combination therapy not only enhanced antitumor efficacy but also converted immunologically “cold” tumors into “hot” tumors, resulting in a several-fold increase in PD-1 inhibitor response rates. In a complementary study, Luo et al. engineered a novel nanoformulation (NP-FeS/GD) containing FeS and a GSDMD plasmid (95). This innovative design simultaneously triggered immunogenic PANoptosis and ferroptosis, eliciting robust innate and adaptive immune responses while effectively reprogramming the immunosuppressive tumor microenvironment, thereby potentiating antitumor immunotherapy.

This section summarizes key research breakthroughs regarding PANoptosis in cancer immunotherapy, focusing on five pivotal aspects (1): modulation of immune cell infiltration and functional states to enhance antitumor immune responses; (2) development of PANoptosis-based combinatorial immunotherapy; (3) establishment of tumor prediction models leveraging PANoptosis molecular signatures; (4) overcoming drug resistance and potentiating immunotherapeutic efficacy through PANoptosis regulation; and (5) nanoengineered strategies synergizing with PANoptosis for antitumor immunotherapy. These advances not only validate the scientific value of PANoptosis in tumor immunotherapy but also demonstrate the remarkable feasibility and innovative potential of targeting PANoptosis to reprogram the TIME.

## 6 Summary and outlook

The discovery of PANoptosis has unveiled a more sophisticated and intricate regulatory framework of programmed cell death. This review introduces the concept and mechanistic basis of PANoptosis, synthesizes existing evidence of its role in tumor progression, and explores its close association with functional and compositional alterations of immune cells within the TIME. The correlation between PANoptosis and TIME highlights its potential as a novel therapeutic target for reprogramming the immunosuppressive microenvironment, thereby advancing cancer immunotherapy. Furthermore, we summarize recent breakthroughs in PANoptosis-based immunotherapies, underscoring its promising clinical applicability and affirming the feasibility and innovation of TIME-targeted strategies. Despite its therapeutic promise, PANoptosis-focused approaches face several challenges:

### 6.1 Complexity of molecular networks

The pathogenic mechanisms of PANoptosis in tumors remain incompletely understood due to its highly interconnected signaling pathways. Emerging technologies—such as single-cell analysis and high-throughput sequencing—may facilitate the identification of PANoptosis-specific molecular signatures and elucidate the crosstalk between upstream regulators and downstream effectors. A deeper mechanistic understanding will enable the development of precision therapies targeting key sensors and executioner molecules.

### 6.2 Lack of Direct and Definitive *in vivo* evidence and clinical validation

Initial research on PANoptosis primarily relied on *in vitro* cell culture models. With advancing studies, animal disease models have provided compelling evidence supporting its pathophysiological significance. Currently, the confirmation of PANoptosis in humans mainly depends on the detection of related molecular markers, as well as transcriptomic and proteomic analyses, while direct and definitive evidence remains limited. Therefore, future research must focus on breakthroughs in human sample analysis techniques and methodologies to accumulate more direct evidence, thereby strengthening the foundation for disease prevention and treatment strategies.

### 6.3 Safety concerns and off-target effects

Therapeutic activation of PANoptosis carries inherent risks, including excessive or aberrant cell death that may exacerbate inflammation, trigger autoimmunity, or cause normal tissue toxicity. A critical challenge lies in achieving a therapeutic window that maximizes tumor cell elimination while minimizing collateral damage to healthy tissues.

### 6.4 Insufficient verification and transformation bottlenecks

Current research on PANoptosis-mediated TIME modulation remains in its infancy. Robust clinical experimental validation is needed to delineate its spatiotemporal regulation and interactions with immune components. Future studies should prioritize mechanistic elucidation and technological integration to facilitate clinical translation of PANoptosis research. By strategically combining PANoptosis with existing therapeutic approaches, researchers may optimize cancer immunotherapy regimens to maximize clinical outcomes.
